# Steroidal and non-steroidal third-generation aromatase inhibitors induce pain-like symptoms via TRPA1

**DOI:** 10.1038/ncomms6736

**Published:** 2014-12-08

**Authors:** Camilla Fusi, Serena Materazzi, Silvia Benemei, Elisabetta Coppi, Gabriela Trevisan, Ilaria M. Marone, Daiana Minocci, Francesco De Logu, Tiziano Tuccinardi, Maria Rosaria Di Tommaso, Tommaso Susini, Gloriano Moneti, Giuseppe Pieraccini, Pierangelo Geppetti, Romina Nassini

**Affiliations:** 1 Department of Health Sciences, Section of Clinical Pharmacology and Oncology, University of Florence, Florence 50139, Italy; 2 Laboratory of Biological and Molecular Biology, Graduate Program in Health Sciences, University of the Extreme South of Santa Catarina (UNESC), Santa Catarina 88806-000, Brazil; 3 Department of Pharmacy, University of Pisa, Pisa 56126, Italy; 4 Department of Health Sciences, Section of Pediatrics, Obstetrics and Gynecology and Nursing, Florence 50139, Italy; 5 Mass Spectrometry Center, University of Florence, Florence 50139, Italy

## Abstract

Use of aromatase inhibitors (AIs), exemestane, letrozole and anastrozole, for breast cancer therapy is associated with severe pain symptoms, the underlying mechanism of which is unknown. The electrophilic nature of AIs suggests that they may target the transient receptor potential ankyrin 1 (TRPA1) channel, a major pathway in pain transmission and neurogenic inflammation. AIs evoke TRPA1-mediated calcium response and current in rodent nociceptors and human cells expressing the recombinant channel. In mice, AIs produce acute nociception, which is exaggerated by pre-exposure to proalgesic stimuli, and, by releasing sensory neuropeptides, neurogenic inflammation in peripheral tissues. AIs also evoke mechanical allodynia and decreased grip strength, which do not undergo desensitization on prolonged AI administration. These effects are markedly attenuated by TRPA1 pharmacological blockade or in TRPA1-deficient mice. TRPA1 is a major mediator of the proinflammatory/proalgesic actions of AIs, thus suggesting TRPA1 antagonists for the treatment of pain symptoms associated with AI use.

## Abstract (web summary)

Use of aromatase inhibitors for breast cancer therapy is associated with severe pain symptoms, the underlying mechanism of which is unknown. Here the authors show that in mice, TRPA1 is a major mediator of the proinflammatory and proalgesic actions of aromatase inhibitors.

## Graphical abstract

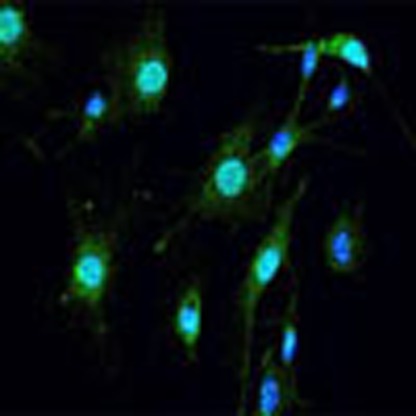

Third-generation aromatase inhibitors (AIs) are currently recommended for adjuvant endocrine treatment as primary, sequential or extended therapy with tamoxifen, for postmenopausal women diagnosed with oestrogen receptor-positive breast cancer^1^,^2^,^3^. AIs include the steroidal exemestane and non-steroidal azole derivatives, letrozole and anastrozole, which, via a covalent (exemestane) and non-covalent (azoles) binding, inactivate aromatase, the enzyme that catalyzes the conversion of androgens to oestrogens in peripheral tissue^4^. The use of AIs is, however, associated with a series of relevant side effects that are reported in 30–60% of treated patients^5^,^6^. Among these, the AI-associated musculoskeletal symptoms (AIMSS) are characterized by morning stiffness and pain of the hands, knees, hips, lower back and shoulders^7^,^8^. In addition to musculoskeletal pain, pain symptoms associated with AIs have recently been more accurately described with the inclusion of neuropathic, diffused and mixed pain^9^. The whole spectrum of painful conditions has been reported to affect up to 40% of patients, and to lead 10–20% of patients to non-adherence or discontinuation of treatment^7^,^8^,^9^,^10^,^11^,^12^,^13^,^14^. Although it has been proposed that oestrogen deprivation and several other factors, including a higher level of anxiety, may contribute to the development of AIMSS and related pain symptoms, none of these hypotheses has been confirmed^9^,^15^. Thus, the exact mechanism of such conditions is still unclear and, consequently, patients are undertreated.

The transient receptor potential ankyrin 1 (TRPA1) channel, belonging to the larger family of the TRP channels^16^,^17^, is a polymodal sensor activated by chemical, mechanical and thermal stimuli^18^,^19^,^20^,^21^,^22^,^23^. TRPA1 is principally expressed by a subpopulation of primary sensory neurons^24^,^25^, which express additional TRPs, including the TRP vanilloid 1 (TRPV1) channel, which is selectively targeted by capsaicin, the hot ingredient of red peppers^16^. TRPA1 and TRPV1 expressing pseudounipolar nociceptors produce and release from central and peripheral terminals the sensory neuropeptides, substance P (SP), neurokinin A (NKA) and calcitonin gene-related peptide (CGRP), which mediate neurogenic inflammation^26^. In particular, TRPA1 is the main target of many different irritant stimuli, such as allyl isothiocyanate (AITC, contained in mustard or wasabi) or cinnamaldehyde (contained in cinnamon), and of an unprecedented series of endogenous reactive molecules produced at sites of inflammation and tissue injury, including reactive oxygen (ROS), nitrative (RNS) or carbonyl (RCS) species^19^,^27^,^28^,^29^,^30^. TRPA1 is emerging as a major nociceptive and hyperalgesic mechanism in a variety of inflammatory pain models such as those induced by formalin, carrageenan and complete Freund adjuvant^31^,^32^,^33^,^34^. Also, in models of neuropathic pain, such as those evoked by spinal nerve ligation^35^, streptozotocin^36^ and chemotherapeutic-induced peripheral neuropathy^37^,^38^,^39^, a key role of TRPA1 has been identified.

The chemical structure of exemestane includes a system of highly electrophilic conjugated Michael acceptor groups, which might react with the thiol groups of reactive cysteine residues^40^. Michael addition reaction with specific cysteine residues is a major mechanism that results in TRPA1 activation by a large variety of electrophilic compounds^19^,^41^,^42^. Aliphatic and aromatic nitriles can react with cysteine to form thiazoline derivatives and accordingly the tear gas 2-chlorobenzylidene malononitrile (CS) has been identified as a TRPA1 agonist^43^. We noticed that both letrozole and anastrozole possess nitrile moieties. Thus, we hypothesized that exemestane, letrozole and anastrozole may produce neurogenic inflammation, nociception and hyperalgesia by targeting TRPA1. Our present findings show that AIs directly stimulate TRPA1, and via this pathway provoke neurogenic inflammatory oedema, acute nociception, mechanical allodynia and reduced grip strength, indicating a new mechanism through which AIs induce cytokine-independent inflammation and pain, and suggesting TRPA1 antagonists as possible innovative therapies for pain-like symptoms associated with the use of AIs.

## Results

### Aromatase inhibitors selectively activate TRPA1 channels

**Figure 1:**
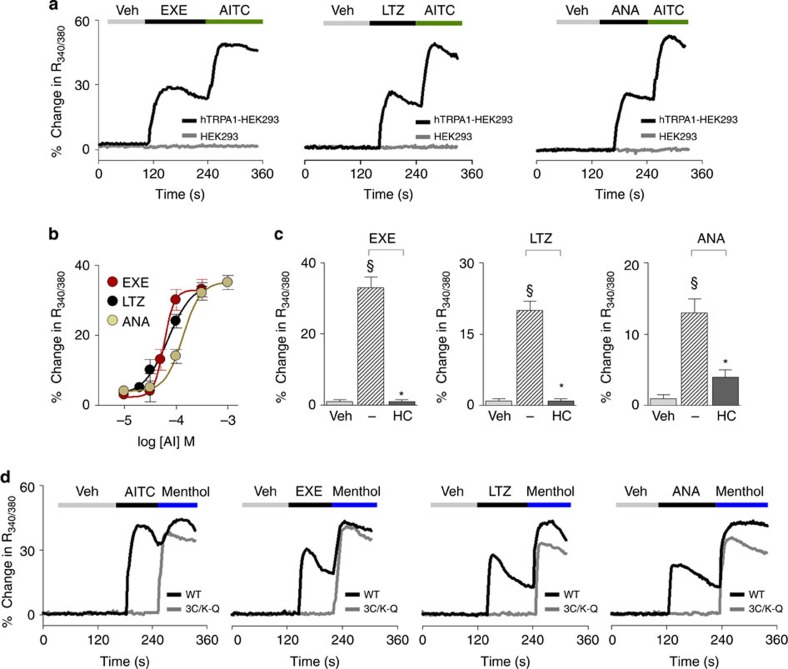
Exemestane (EXE), letrozole (LTZ) and anastrozole (ANA) selectively activate the human TRPA1 channel. (**a**) Representative traces of intracellular calcium response evoked by the aromatase inhibitors (AIs), EXE (100 μM), LTZ (100 μM) and ANA (100 μM), in HEK293 cells transfected with the cDNA for human TRPA1 (hTRPA1-HEK293), which respond to the selective TRPA1 agonist, allyl isothiocyanate (AITC; 30 μM). AITC (30 μM), EXE, LTZ and ANA (all 100 μM) fail to produce any calcium response in untransfected-HEK293 cells (HEK293). (**b**) Concentration-response curves to EXE, LTZ and ANA, yielded EC_50_ (95% confidence interval) of 58 (46–72) μM, 69 (43–109) μM, and 134 (96–186) μM, respectively. (**c**) AI-evoked calcium response in hTRPA1-HEK293 is abolished by the selective TRPA1 antagonist, HC-030031 (HC; 30 μM). (**d**) Representative traces of cells transfected with the cDNA codifying for the mutant hTRPA1 channel (3C/K-Q), which are insensitive to AITC (30 μM) or AIs (100 μM), but respond to the non-electrophilic agonist, menthol (100 μM), whereas HEK293 cells transfected with the cDNA codifying for wild-type hTRPA1 (WT) respond to all the drugs. Veh is the vehicle of AIs; dash (−) indicates the vehicle of HC. Each point or column represents the mean±s.e.m. of at least 25 cells from 3–6 independent experiments. ^§^*P*<0.05 versus Veh, *P<0.05 versus EXE, LTZ or ANA group; ANOVA and Bonferroni *post hoc* test.

To explore whether AIs gate the human TRPA1 channel, we first used cells stably transfected with human TRPA1 cDNA (hTRPA1-HEK293). In hTRPA1-HEK293 cells, which respond to the selective TRPA1 agonist AITC (30 μM), but not in untransfected HEK293 cells, the three AIs, exemestane, letrozole and anastrozole, evoked concentration-dependent calcium responses that were inhibited by the selective TRPA1 antagonist, HC-030031 (30 μM)^44^ (Fig. 1a–c). EC_50_ of AIs ranged between 58 and 134 μM (Fig. 1b). The calcium response was abated in a calcium-free medium, thus supporting the hypothesis that the increase in intracellular calcium originates from extracellular sources (Supplementary Fig. 1a). In HEK293 cells stably transfected with human TRPV1 cDNA (hTRPV1-HEK293) all AIs (100 μM) were ineffective (Supplementary Fig. 1b). Key amino-acid residues are required for channel activation by electrophilic TRPA1 agonists^19^,^41^,^42^. Notably, HEK293 cells expressing a mutated TRPA1 channel (3C/K-Q), which presents substitutions of three cysteine with serine (C619S, C639S, C663S) and one lysine with glutamine (K708Q) residues, were insensitive to both AITC^19^,^41^ and all three AIs, while maintaining sensitivity to the non-electrophilic agonists, menthol^29^ or icilin^42^ (Fig. 1d and Supplementary Fig. 1c). This finding supports the hypothesis that the ability of AIs to target TRPA1 derives from their electrophilic nature. Electrophysiology experiments recapitulated findings obtained by means of the calcium assay. Exemestane, letrozole and anastrozole selectively activated a concentration-dependent inward current in hTRPA1-HEK293 cells, a response that was abated by HC-030031 (Supplementary Fig. 1d). AIs did not evoke any current in untransfected HEK293 cells (Supplementary Fig. 1d).

**Figure 2:**
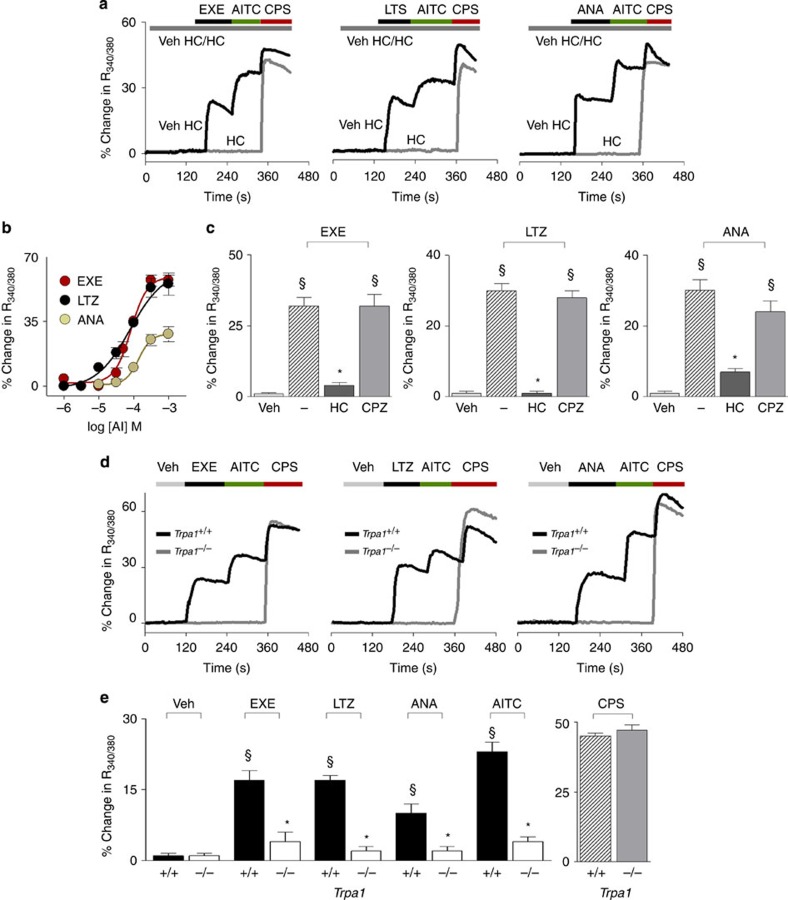
Exemestane (EXE), letrozole (LTZ) and anastrozole (ANA) selectively activate the native TRPA1 channel expressed in rodent dorsal root ganglion (DRG) neurons. (**a**) Representative traces of calcium response evoked by EXE (100 μM), LTZ (100 μM), ANA (300 μM) in cultured rat DRG neurons, which also respond to allyl isothiocyanate (AITC; 30 μM) and capsaicin (CPS; 0.1 μM). Calcium responses evoked by AIs and AITC are abolished by the selective TRPA1 antagonist, HC-030031 (HC; 30 μM), which does not affect response to CPS. (**b**) Concentration-response curves of EXE, LTZ and ANA, yielded EC_50_ (95% confidence interval) of 82 (61–108) μM, 78 (39–152)  μM, and 135 (78–231)  μM, respectively. (**c**) Calcium responses induced by AIs are inhibited by HC and unaffected by the TRPV1 antagonist, capsazepine (CPZ; 10 μM). ^§^*P*<0.05 versus Veh, *P<0.05 versus EXE, LTZ or ANA; ANOVA and Bonferroni *post hoc* test. (**d**) Representative traces and (**e**) pooled data of the calcium response evoked by EXE, LTZ, ANA (all 100 μM) or AITC (30 μM), in neurons isolated from *Trpa1*^*+/+*^ mice. Neurons isolated from *Trpa1*^*−/−*^ mice do not respond to AITC, EXE, LTZ and ANA, whereas they do respond normally to CPS (0.1 μM). In DRG neurons isolated from both *Trpa1*^*+/+*^ and *Trpa1*^*−/−*^ mice, calcium response is evaluated only in capsaicin responding neurons. ^§^*P*<0.05 versus Veh, *P<0.05 versus EXE, LTZ, ANA or AITC-*Trpa1*^*+/+*^, ANOVA and Bonferroni *post hoc* test. Veh is the vehicle of AIs; dash (-) indicates the combination of the vehicles of HC and CPZ. Each point or column represents the mean±s.e.m. of at least 25 neurons obtained from 3 to 7 independent experiments.

Next, to verify whether exemestane, letrozole and anastrozole stimulate nociceptive sensory neurons via TRPA1 activation, we used primary culture of both rat and mouse dorsal root ganglion (DRG) neurons. Similar to AITC^19^, all AIs produced a concentration-dependent calcium response (Fig. 2a,b) in a proportion (about 30%) of cells that responded to the selective TRPV1 agonist, capsaicin (0.1 μM). All cells responding to AIs, but none of the non-responding cells, invariably responded to a subsequent high concentration of AITC (30 μM) (Fig. 2a), further documenting TRPA1 as the target of AIs. In rat DRG neurons, EC_50_ ranged between 78 and 135 μM (Fig. 2b). Calcium responses evoked by the three AIs were abated by HC-030031 (30 μM), but were unaffected by the selective TRPV1 antagonist, capsazepine (10 μM) (Fig. 2c). Notably, AITC and all AIs produced a calcium response in capsaicin-sensitive DRG neurons isolated from wild-type (*Trpa1*^*+/+*^) mice, an effect that was absent in neurons obtained from TRPA1-deficient (*Trpa1*^*−/−*^) mice (Typical traces Fig. 2d and pooled data Fig. 2e).

### AIs activate nociceptive and hyperalgesic TRPA1-dependent pathways

It has been well documented that local exposure to TRPA1 agonists in experimental animals is associated with an immediate nociceptive response, lasting for a few minutes, and a delayed more prolonged mechanical allodynia^18^,^19^. To investigate whether AIs activate such a nociceptive and hyperalgesic TRPA1-dependent pathway, we used one steroidal (exemestane) and one non-steroidal (letrozole) AI. Given the chemical similarity and the hypothesized analogous mechanism of the two non-steroidal AIs, to minimize the number of animals used, anastrozole was not investigated in the following *in vivo* experiments. Intraplantar (i.pl.) injection (20 μl per paw) of exemestane (1, 5 and 10 nmol) (Supplementary Fig. 2a) or letrozole (10, 20 nmol) (Supplementary Fig. 2e) evoked an acute (0–5 min) nociceptive response and a delayed (15–120 min for exemestane and 15–240 min for letrozole) mechanical allodynia in C57BL/6 mice (Supplementary Fig. 2c,g). Both the nociceptive response and mechanical allodynia evoked by AIs were confined to the treated paw (Supplementary Fig. 2c,g) and were almost completely prevented by intraperitoneal (i.p.) pretreatment with HC-030031 (100 mg kg^−1^), but not with capsazepine (4 mg kg^−1^) (Supplementary Fig. 2b,d,f,h). Furthermore, similar to results obtained in C57BL/6 mice, local injection (i.pl.) of exemestane or letrozole in *Trpa1*^*+/+*^ mice evoked an early nociceptive response and a delayed mechanical allodynia (Supplementary Fig. 2i,j). *Trpa1*^*−/−*^ mice did not develop such responses (Supplementary Fig. 2i,j). Thus, by using both pharmacological and genetic tools, we demonstrated that local administration of both steroidal and non-steroidal AIs produces a typical TRPA1-dependent behaviour, characterized by acute nociception and delayed mechanical allodynia.

### AIs produce neurogenic oedema by releasing sensory neuropeptides

**Figure 3:**
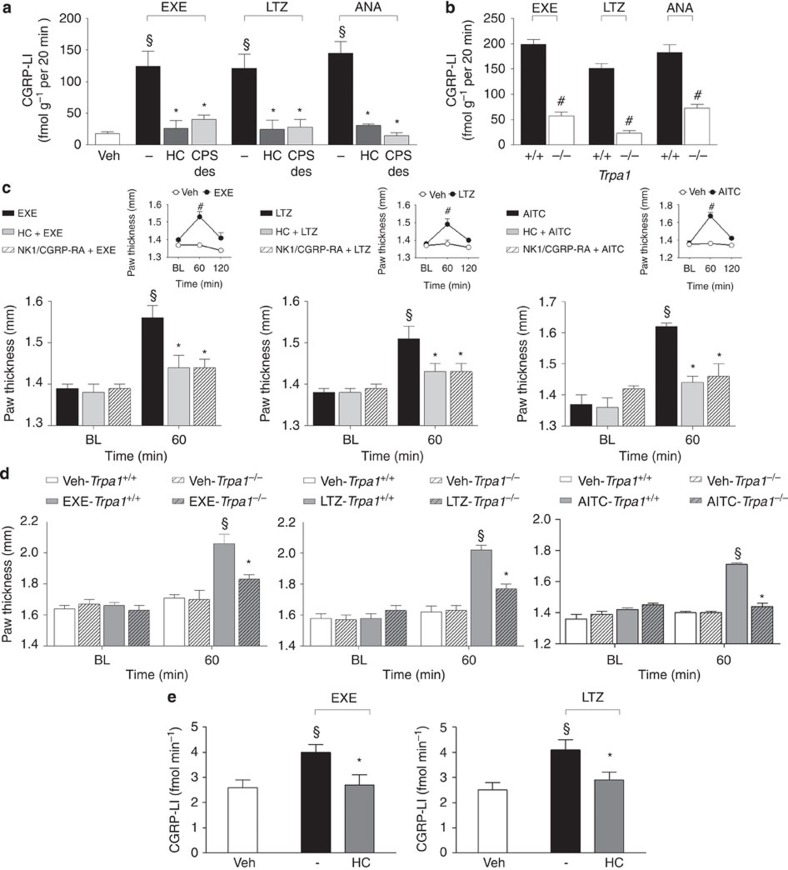
Aromatase inhibitors release calcitonin gene-related peptide (CGRP) and produce neurogenic edema. (**a**) Exemestane (EXE), letrozole (LTZ) and anastrozole (ANA) (all 100 μM) increase the CGRP-like immunoreactivity (CGRP-LI) outflow from slices of rat dorsal spinal cord. This effect is prevented by HC-030031 (HC; 30 μM) or after exposure to capsaicin (10 μM, 20 min; CPS-des). (**b**) EXE, LTZ and ANA (all 100 μM) increase the CGRP-LI outflow from spinal cord slices obtained from *Trpa1*^*+/+*^, but not from *Trpa1*^*−/−*^ mice. Results are mean±s.e.m. of at least four independent experiments. Veh is the vehicle of EXE, LTZ and ANA, dash (-) indicates the vehicle of HC and CPS. ^§^*P*<0.05 versus Veh, *P<0.05 versus EXE, LTZ or ANA; ANOVA followed by Bonferroni *post hoc* test. ^#^*P*<0.05 versus EXE-, LTZ-, ANA-*Trpa1*^*+/+*^, Student’s *t*-test. (**c**) In C57BL/6 mice intraplantar (i.pl.) injection (20 μl) of EXE (10 nmol), LTZ (20 nmol) or allyl isothiocyanate (AITC; 10 nmol) induces paw oedema, which peaks at 60 min and fades 120 min after injection (**c**, upper insets), and is attenuated by pretreatment with HC (100 mg kg^−1^ intraperitoneal, i.p.) or the combination of the selective antagonists of the neurokinin-1 receptor, (NK1-RA), L-733,060, and of the CGRP receptor (CGRP-RA), CGRP8-37, (both, 2 μmol kg^−1^, intravenous). (**d**) Paw oedema induced by EXE, LTZ and AITC (i.pl.) in *Trpa1*^*+/+*^ mice is markedly reduced in *Trpa1*^*−/−*^ mice. BL=baseline level. Results are mean±s.e.m. of at least five mice for each group. Veh is the vehicle of EXE, LTZ and AITC. ^#^*P*<0.05 versus Veh, Student’s *t*-test; ^§^*P*<0.05 versus BL values, *P<0.05 versus EXE, LTZ, AITC or EXE-, LTZ-, AITC-*Trpa1*^*+/+*^; ANOVA followed by Bonferroni *post hoc* test. (**e**) Injection (50 μl) of EXE (5 nmol) or LTZ (10 nmol) in the rat knee increases CGRP-LI levels in the synovial fluid, an effect that is markedly attenuated by pretreatment with HC (100 mg kg^−1^, i.p.). Results are mean±s.e.m. of at least five mice for each group. Veh is the vehicle of EXE and LTZ, dash (-) indicates the vehicle of HC. ^§^*P*<0.05 versus Veh, *P<0.05 versus EXE, LTZ; ANOVA followed by Bonferroni *post hoc* test.

TRPA1 is expressed by peptidergic nociceptors, and its stimulation is associated with proinflammatory neuropeptide release and the ensuing neurogenic inflammatory responses^19^,^45^. First, we explored whether AIs are able to directly promote the release of CGRP (one of the proinflammatory neuropeptides, which are usually co-released on stimulation of peptidergic nociceptors)^26^,^46^ via a TRPA1-dependent pathway. AIs increased CGRP outflow from slices of rat dorsal spinal cord (an anatomical area enriched with central terminals of nociceptors). This effect was substantially attenuated in rat slices pretreated with a desensitizing concentration of capsaicin (10 μM, 20 min) or in the presence of HC-030031 (Fig. 3a). The increase in CGRP outflow observed in slices obtained from *Trpa1*^*+/+*^ mice was markedly reduced in slices obtained from *Trpa1*^*−/−*^ mice (Fig. 3b).

These neurochemical data were corroborated by functional experiments. Injection (i.pl.) of the TRPA1 agonist, AITC (10 nmol per paw), induced paw oedema that peaked at 60 min after injection. The response was abated by treatment with HC-030031 (100 mg kg^−1^, i.p.) or a combination of the SP neurokinin-1 (NK-1) receptor antagonist, L-733,060, and the CGRP receptor antagonist, CGRP8-37 (both, 2 μmol kg^−1^, intravenous, i.v.) (Fig. 3c). Similarly, we found that i.pl. administration of exemestane (10 nmol per paw) and letrozole (20 nmol per paw) caused paw oedema that peaked at 60 min and faded 120 min after injection (Fig. 3c, insets). Treatment with HC-030031 (100 mg kg^−1^, i.p.) or a combination of L-733,060 and CGRP8-37 (both, 2 μmol kg^−1^, i.v.), markedly reduced the AI-evoked oedema (Fig. 3c). No oedema was found in the paw contralateral to that injected with AIs (Supplementary Fig. 2k). Importantly, the oedema produced in *Trpa1*^*+/+*^ mice by exemestane and letrozole was markedly attenuated in *Trpa1*^*−/−*^ mice (Fig. 3d). Next, to directly evaluate the ability of AIs to release CGRP from peripheral terminals of peptidergic nociceptors, AIs were administered to the rat knee joint. Intra-articular (i.a., 50 μl) injection of exemestane (5 nmol) or letrozole (10 nmol) increased CGRP levels in the synovial fluid, an effect that was markedly attenuated by pretreatment with HC-030031 (100 mg kg^−1^, i.p.) (Fig. 3e). Neurochemical and functional data indicate that AIs by TRPA1 activation release sensory neuropeptides from sensory nerve endings, and by this mechanism promote neurogenic inflammatory responses in the innervated peripheral tissue.

### Systemic AIs induce prolonged pain-like effects by targeting TRPA1

**Figure 4:**
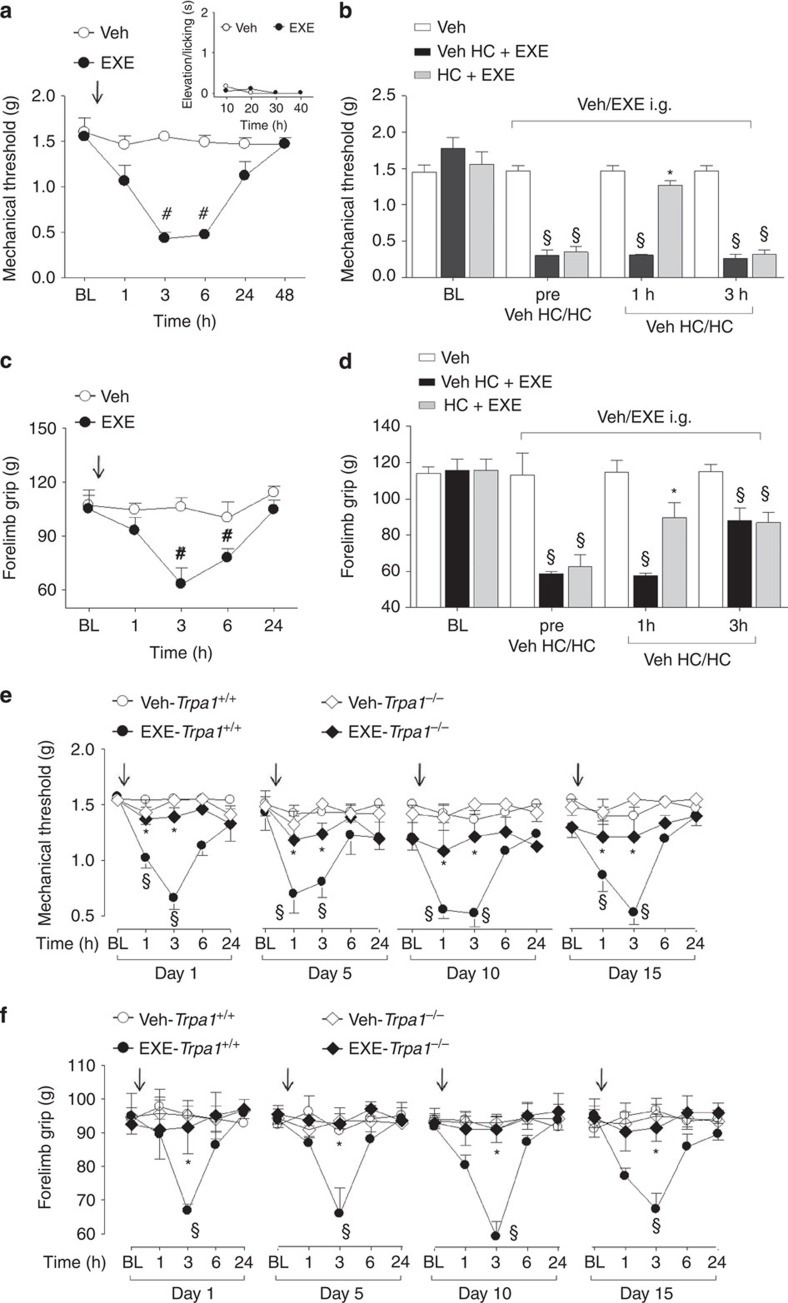
Intragastric exemestane (EXE) induces TRPA1-dependent prolonged mechanical allodynia and reduction in forelimb grip strength in mice. In C57BL/6 mice intragastric (i.g.) administration of EXE (10 mg kg^−1^) induces (**a**) mechanical allodynia and (**c**) a reduction in forelimb grip strength that last 3–6 h after administration. EXE does not produce any acute nocifensor behaviour as measured by the indicated test (**a**, inset). (**b**,**d**) Three hours after EXE administration, HC-030031 (HC; 100 mg kg^−1^ i.p.) reverts both mechanical allodynia and the reduction in forelimb grip strength. HC inhibition is no longer visible 3 h after its administration. Veh is the vehicle of EXE. ^#^*P*<0.05 versus Veh; Student’s *t*-test (**a**,**c**) and ^§^*P*<0.05 versus Veh and *P<0.05 versus Veh HC-EXE; ANOVA followed by Bonferroni *post hoc* test (**b**,**d**). (**e**,**f**) EXE (once a day for 15 consecutive days, 10 mg kg^−1^ i.g.) induces reproducible mechanical allodynia and decrease in forelimb grip strength at day 1, 5, 10 and 15 in *Trpa1*^*+/+*^mice. Arrows indicate Veh or EXE administration. Both these effects are markedly reduced in *Trpa1*^*−/−*^ mice. ^§^*P*<0.05 versus Veh-*Trpa1*^*+/+*^, *P<0.05 versus EXE-*Trpa1*^*+/+*^; ANOVA followed by Bonferroni *post hoc* test. Results are mean±s.e.m. of at least five mice for each group. In all conditions, baseline (BL) levels were recorded 30 min before EXE administration.

**Figure 5:**
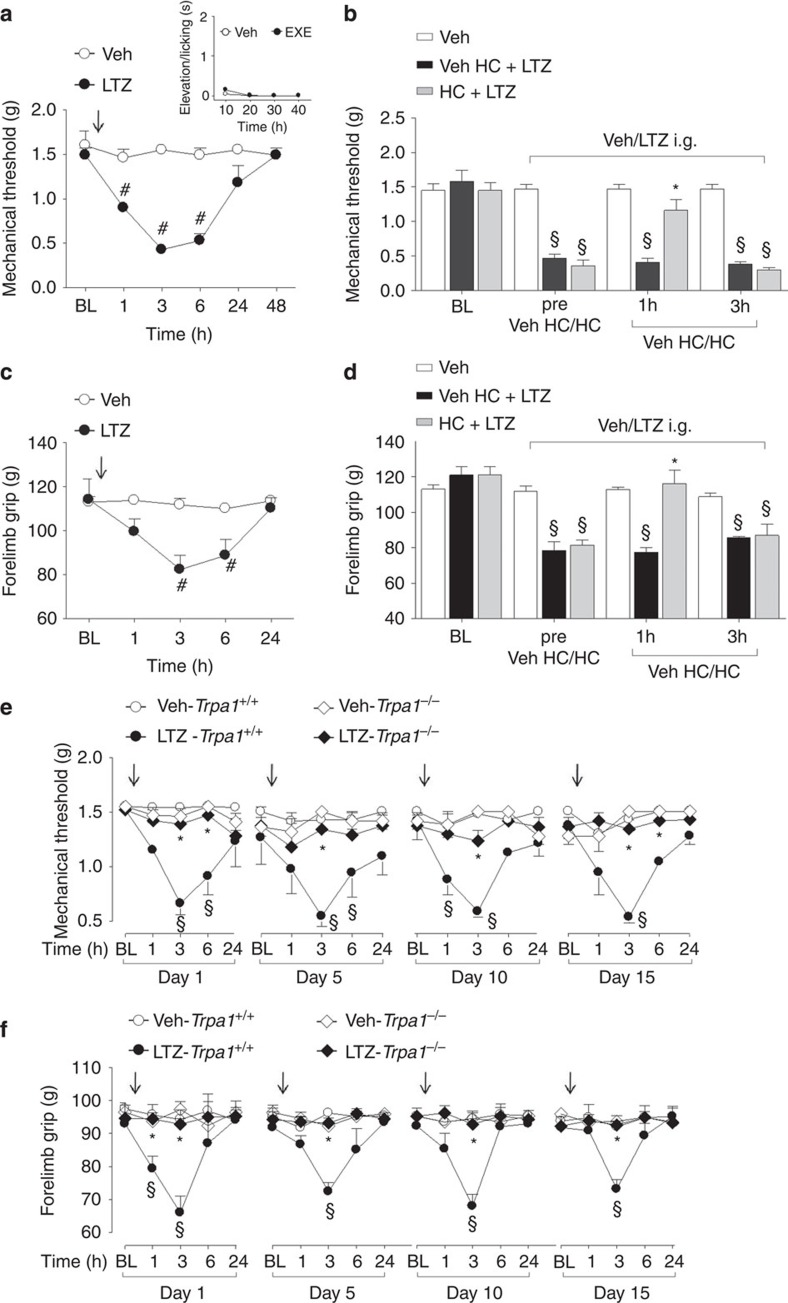
Intragastric letrozole (LTZ) induces TRPA1-dependent prolonged mechanical allodynia and reduction in forelimb grip strength in mice. In C57BL/6 mice intragastric (i.g.) administration of LTZ (0.5 mg kg^−1^) induces (**a**) mechanical allodynia and (**c**) reduction in forelimb grip strength that last 3–6 h after administration. LTZ does not produce any acute nocifensor behaviour as measured by the indicated test (**a**, inset). (**b**,**d**) Three hours after LTZ administration, HC-030031 (HC; 100 mg kg^−1^ i.p.) reverts both mechanical allodynia and the reduction in forelimb grip strength. HC inhibition is no longer visible 3 h after its administration. Veh is the vehicle of LTZ. ^#^*P*<0.05 versus Veh; Student’s *t*-test (**a**,**c**) and ^§^*P*<0.05 versus Veh and *P<0.05 versus Veh HC-LTZ; ANOVA followed by Bonferroni *post hoc* test (**b**,**d**). (**e**,**f**) LTZ (once a day for 15 consecutive days, 0.5 mg kg^−1^ i.g.) induces reproducible mechanical allodynia and decrease in forelimb grip strength at day 1, 5, 10 and 15 in *Trpa1*^*+/+*^mice. Arrows indicate Veh or LTZ administration. Both effects are markedly reduced in *Trpa1*^*−/−*^ mice. ^§^*P*<0.05 versus Veh-*Trpa1*^*+/+*^, *P<0.05 versus LTZ-*Trpa1*^*+/+*^; ANOVA followed by Bonferroni *post hoc* test. Results are mean±s.e.m. of at least five mice for each group. In all conditions baseline (BL) levels were recorded 30 min before LTZ administration.

AIs are given to patients by a systemic route of administration. Therefore, we explored in mice whether intraperitoneal (i.p.) or intragastric (i.g.) administration of exemestane and letrozole could produce pain-like effects via TRPA1 activation. For i.p. administration experiments, doses, corresponding to those used in humans, were selected according to the mouse to human conversion factor indicated by the National Institute of Health^47^. Exemestane (5 mg kg^−1^, i.p.) or letrozole (0.5 mg kg^−1^, i.p) injection did not produce any visible nociceptive behaviour (Supplementary Figs 3a,4a, insets) in mice. However, 3 h after exemestane or letrozole administration, mice developed a prolonged (3 h) mechanical allodynia (Supplementary Figs 3a,4a) and a reduction in forelimb grip strength (Supplementary Figs 3c,4c), a test used in its clinical version for the study of musculoskeletal pain in patients^48^. When mechanical allodynia by exemestane or letrozole was at its maximum, systemic HC-030031 administration (100 mg kg^−1^, i.p.) transiently reverted both responses (Supplementary Figs 3b,d and 4b,d). Furthermore, mechanical allodynia and the reduction in forelimb grip strength produced by exemestane and letrozole in *Trpa1*^*+/+*^ mice were markedly reduced in *Trpa1*^*−/−*^ mice (Supplementary Figs 3e,f and 4e,f). In experiments where AIs were given by intragastric (i.g.) gavage, doses were adjusted considering the oral bioavailability in humans, which is 99% for letrozole^49^, and 40% (with food) for exemestane^50^. First, we found that after i.g. administration of exemestane (10 mg kg^−1^) or letrozole (0.5 mg kg^−1^) their peak plasma levels (13.2±1.7 ng ml^−1^, *n*=5; and 60.5±12.1 ng ml^−1^, *n*=5, respectively, Supplementary Fig. 5) approximated the maximum plasma concentrations found in humans^49^,^51^. Second, results similar to those obtained after i.p. administration were reported when AIs were given by i.g. gavage. First, exemestane (10 mg kg^−1^, i.g.) or letrozole (0.5 mg kg^−1^, i.g.) ingestion was not associated with any spontaneous nocifensor behaviour (Figs 4a, 5a, insets). Second, exemestane or letrozole produced, with a similar time-course, mechanical allodynia and a marked reduction in forelimb grip strength (Figs 4a,c and 5a,c). Pretreatment with HC-030031 or deletion of TRPA1 (*Trpa1*^*−/−*^ mice) significantly attenuated both responses (Figs 4b,d,e,f and 5b,d,e,f).

Furthermore, since in clinical practice patients are treated with AIs on a daily basis over very long periods of time (up to 5 years), we asked whether exemestane or letrozole maintains the ability to evoke a TRPA1-dependent mechanical hypersensitivity and decreased grip strength on repeated administration. In *Trpa1*^*+/+*^ mice, treatment with systemic exemestane (5 mg kg^−1^, i.p.) or letrozole (0.5 mg kg^−1^ i.p) (both once a day for 15 consecutive days) produced at day 1, 5, 10 and 15 a transient (from 1 to 6 h) and reproducible mechanical allodynia (Supplementary Figs 3e, 4e). Importantly, in *Trpa1*^*−/−*^ the proalgesic action of AIs was markedly attenuated (Supplementary Figs 3e, 4e). In addition, the decrease in the grip strength was maintained, without undergoing desensitization, over the entire time period of daily i.p. administration of exemestane or letrozole (Supplementary Figs 3f,4f). Both these effects of AIs were significantly reduced in *Trpa1*^*−/−*^ mice (Supplementary Figs 3f, 4f). Similar results were obtained after i.g. administration of exemestane or letrozole (once a day for 15 consecutive days at the dose of 10 mg kg^−1^ i.g. or 0.5 mg kg^−1^ i.g., respectively). Both mechanical allodynia and decreased grip strength were observed, without signs of desensitization, over the 15 days of observation in *Trpa1*^*+/+*^ mice, but were markedly reduced in *Trpa1*^*−/−*^ mice (Figs 4e,f and 5e,f). Altogether, the present data demonstrate that both steroidal and non-steroidal third-generation AIs induce a series of pain-like effects predominantly via a TRPA1-dependent mechanism, effects that over time do not undergo desensitization, thus mimicking the chronic clinical condition.

### AI-evoked TRPA1 activation is enhanced by proinflammatory stimuli

**Figure 6:**
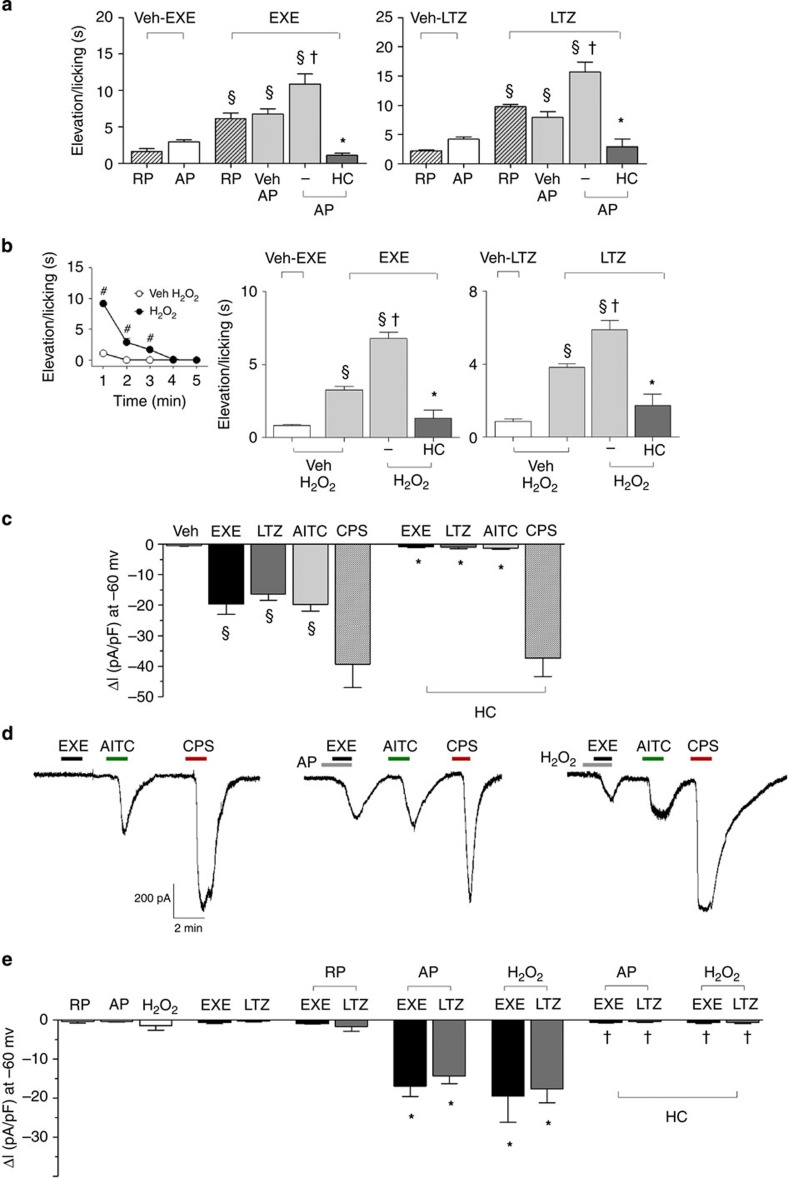
TRPA1 activation by exemestane (EXE) and letrozole (LTZ) is enhanced by proinflammatory stimuli. (**a**) Intraplantar (i.pl.; 10 μl) pretreatment (10 min) with the proteinase-activated receptor 2 (PAR2) activating peptide (AP; 1 μg), but not with the inactive PAR2 reverse peptide (RP; 1 μg), enhances nocifensor behaviour produced by EXE (1 nmol per 10 μl, i.pl.) or LTZ (10 nmol per 10 μl, i.pl.). AP and RP alone causes negligible nociception. The potentiated responses to EXE or LTZ are markedly attenuated by HC-030031 (HC; 100 mg kg^−1^, i.p.). (**b**) H_2_O_2_ (0.5 μmol per 10 μl, i.pl.) injection produces a transient nocifensor behaviour, lasting only 5 min (**b**, inset). Pretreatment (10 min before AI administration) with H_2_O_2_ (0.5 μmol per 10 μl, i.pl.) increases nocifensor behaviour produced by EXE (1 nmol per 10 μl, i.pl.) or LTZ (10 nmol per 10 μl, i.pl.). HC (100 mg kg^−1^, i.p.) inhibits the exaggerated responses to both EXE and LTZ. Dash (-) indicates the vehicle of HC. Points or columns are mean±s.e.m. of at least fiv mice for each group. ^§^*P*<0.05 versus RP or AP or Veh H_2_O_2_; ^†^*P*<0.05 versus Veh AP/EXE or Veh AP/LTZ or Veh H_2_O_2_/EXE or Veh H_2_O_2_/LTZ; *P<0.05 versus AP/EXE or AP/LTZ or H_2_O_2_/EXE or H_2_O_2_/LTZ; ANOVA followed by Bonferroni *post hoc* test. ^#^*P*<0.05 versus Veh H_2_O_2_, Student’s *t*-test. (**c**) An active concentration of EXE or LTZ (both 100 μM) evokes inward currents in rat dorsal root ganglion (DRG) neurons, which also respond to allyl isothiocyanate (AITC; 100 μM) and capsaicin (CPS; 1 μM). Inward currents evoked by EXE, LTZ or AITC are inhibited in the presence of HC (50 μM), which does not affect CPS-evoked currents. Typical traces (**d**) and pooled data (**e**) showing that pre-exposure to AP (100 μM) or H_2_O_2_ (100 μM) exaggerates currents evoked by a subthreshold concentration of EXE and LTZ (both 20 μM). The inactive RP does not affect responses to EXE or LTZ (both 20 μM). The potentiated responses to EXE or LTZ are markedly attenuated by HC (50 μM). Veh is the vehicle of EXE, LTZ and AITC. Results are mean±s.e.m. of at least five independent experiments. ^§^*P*<0.05 versus Veh, *P<0.05 versus EXE, LTZ or AITC and ^†^*P*<0.05 versus EXE- or LTZ-AP and EXE- or LTZ-H_2_O_2_; ANOVA followed by Bonferroni *post hoc* test.

Although it affects a large proportion of subjects, not all patients treated with AIs develop AIMSS. One possible explanation for the peculiar susceptibility to AIMSS of some patients is that, if TRPA1 activation is a necessary prerequisite, *per se* it is not sufficient, and additional proalgesic factors must contribute to the development of pain symptoms. It has been reported that stimulation of proalgesic pathways exaggerates TRPA1-dependent responses *in vitro* and *in vivo*^52^,^53^. One example of such potentiating action has been reported for the proteinase-activated receptor-2 (PAR2), whose subthreshold activation results in an exaggerated response to the TRPA1 agonist, AITC^52^. PAR2 undergoes activation on a unique proteolytic mechanism by cleavage of its tethered ligand domain by trypsin and other proteases, thus mediating inflammation and hyperalgesia^54^. On this basis, and following a previously reported protocol^52^, we explored, by *in vivo* functional experiments in C57BL/6 mice, whether PAR2 activation exaggerates TRPA1-dependent hypersensitivity induced by AIs. Before (10 min) injection (i.pl.) of the PAR2-activating peptide (AP) (PAR2-AP, 1 μg per paw), but not the reverse peptide (RP) (PAR2-RP, 1 μg per paw, inactive on PAR2), markedly enhanced the duration of licks and flinches of the hind paw produced by local injection (i.pl.) of exemestane (1 nmol per paw) and letrozole (10 nmol per paw) (Fig. 6a). The injected dose of PAR2-AP, as well as PAR2-RP, did not cause *per se* any visible acute nocifensor response (Fig. 6a). The exaggerated responses to the combination of PAR2-AP and exemestane or letrozole were inhibited by HC-030031 (100 mg kg^−1^, i.p.) (Fig. 6a).

We also tested the ability of a recognized endogenous TRPA1 agonist, H_2_O_2_ (refs 27, 28) to increase the nocifensor response of exemestane or letrozole. In addition, we explored the ability of AIs to increase either nociception or mechanical allodynia to H_2_O_2_. H_2_O_2_ (0.5 μmol per paw) injection produced a transient nocifensor behaviour that terminated within 5 min (Fig. 6b, inset). We found that 10 min after H_2_O_2_ injection (when baseline levels of nociception were restored) administration of exemestane (1 nmol per paw) and letrozole (10 nmol per paw) evoked nociceptive responses markedly increased as compared with vehicle-pretreated mice (Fig. 6b). The exaggerated responses to AIs were inhibited by HC-030031 (Fig. 6b). Thus, both homologous activation of the channel by the TRPA1 agonist H_2_O_2_, or heterologous stimulation of a classical proinflammatory pathway, such as PAR2, converge in a final common pathway, which results in the potentiation of the AI-evoked and TRPA1-dependent proalgesic mechanism. In the attempt to understand the mechanism underlying the *in vivo* potentiation between PAR2 or H_2_O_2_ and AIs, cultured DRG neurons were challenged with combinations of these same agents. First, in *in vitro* electrophysiological experiments, we found that AITC, exemestane and letrozole (all 100 μM) produced inward currents in cultured DRG neurons, effects that were abated in the presence of HC-030031 (50 μM). However, HC-030031 did not affect the inward current produced by capsaicin (Fig. 6c). Second, we showed that pre-exposure to subthreshold concentrations of PAR2-AP or H_2_O_2_ enhanced currents evoked by subthreshold concentrations of either exemestane or letrozole (both 20 μM) (Typical traces Fig. 6d and pooled data Fig. 6e). Third, HC-030031 inhibited the exaggerated responses (Fig. 6e).

## Discussion

In the present study, we provide for the first time evidence that third-generation steroidal and non-steroidal AIs, proven to be very effective drugs in the treatment of hormone receptor-positive breast cancer^1^,^2^, selectively target the TRPA1 channel. This conclusion derives from a series of experiments in cells expressing the recombinant human TRPA1 or in rodent DRG neurons expressing the native channel. Indeed, calcium responses and currents evoked by AIs are confined to TRPA1-expressing cells, and are selectively abolished by HC-030031, or absent in neurons obtained from TRPA1-deficient mice. Exemestane exhibits a chemical structure with a system of highly electrophilic conjugated Michael acceptor groups^40^. A variety of known TRPA1 agonists, including acrolein and other α,β-unsaturated aldehydes, possesses an electrophilic carbon or sulfur atom that is subject to nucleophilic attack (Michael addition) by cysteine and lysine residues^55^. Nitriles also exhibit electrophilic properties^56^, which may result in TRPA1 gating^43^. Non-steroidal letrozole and anastrozole possess nitrile moieties that underscore their potential ability to activate TRPA1. We show that key cysteine and lysine residues, required for channel activation by electrophilic agonists^19^,^41^,^42^, are also required for TRPA1 activation by AIs. Thus, the three AIs, most likely because of their electrophilic nature, selectively target TRPA1, whereas TRPV1, TRPV2, TRPV3 and TRPV4 all co-expressed with TRPA1 (refs 24, 25), and other channels or receptors in DRG neurons do not seem to play a relevant role in the direct excitation of nociceptors by AIs.

TRPA1-expressing neurons activated by AIs also responded to capsaicin, a selective TRPV1 agonist. As TRPV1 is considered a specific marker of nociceptors^57^, AIs may be assumed to activate pain-like responses. *In vivo* stimulation of the irritant TRPA1 receptor in rodents produces an early nociceptive behaviour, followed by a delayed and prolonged mechanical allodynia^18^,^19^,^44^. Subcutaneous exemestane and letrozole recapitulated the two effects produced by TRPA1 agonists and produced such responses in a TRPA1-dependent way.

Magnetic resonance imaging of painful wrists in patients treated with AIs has shown signs of inflammatory tenosynovitis poorly reverted by common anti-inflammatory treatments^12^. Systemic increases in plasma cytokines have not been found in patients with AIMSS and, therefore, do not appear to represent the underlying mechanism for such inflammatory conditions^9^,^13^. This implies that pathways different from cytokine-dependent inflammation operate in joints of patients treated with AIs. As TRPA1 is expressed by a subpopulation of peptidergic nociceptors, which mediate neurogenic inflammation^24^,^25^,^26^, we anticipated that AIs, by targeting TRPA1, release proinflammatory neuropeptides, thereby causing neurogenic plasma extravasation. Pharmacological and genetic findings indicate that AIs produce a specific type of edema, which is neurogenic in nature. The conclusion is corroborated by the direct neurochemical observation that exemestane and letrozole evoke TRPA1-dependent CGRP release from peripheral endings of primary sensory neurons. The neurogenic component, mediated by TRPA1-activation and sensory neuropeptide release, may thus represent an important mechanism contributing to the cytokine-independent inflammation observed in AI users.

When AIs were given to mice by systemic (intraperitoneal or intragastric) administration, no acute nocifensive response was observed, but, after ~1 h delay they produced a prolonged condition (up to 6 h) of mechanical allodynia and a decrease in forelimb grip strength. Also, in this case, pharmacological and genetic results indicate that AI-evoked pain-like responses are principally TRPA1-dependent. In clinical practice, AIs are used for a 3- or 5-year period, and the pain condition associated with their use is often persistent^58^. Although the present experimental conditions can not fully mimic the clinical setting in cancer patients, our findings suggest that the TRPA1-dependent ability of AIs to produce mechanical allodynia and to decrease forelimb grip strength is maintained and does not undergo desensitization in mice over a time period of 15 days, which broadly corresponds to a 1-year time in humans. Despite a general good tolerability^11^, AIs produce some types of pain, including AIMSS and neuropathic, diffuse and mixed pain in 10–20% of the treated patients^9^. The reason why only some of the patients exposed to AIs develop these severe pain conditions, which may lead to non-adherence or therapy discontinuation, is unknown.

Here, we reveal the key role of TRPA1 as the main mediator of exemestane- and letrozole-evoked nociceptor stimulation. However, it is likely that additional factors contribute to determine the development of AIMSS and related pain symptoms, particularly in those susceptible patients who suffer from the more severe form of this adverse reaction. *In vitro* and *in vivo* experiments with the co-administration of AIs and pro-algesic stimuli, such as PAR2-AP, an agonist of the pro-inflammatory receptor, PAR2, and the TRPA1 agonist, H_2_O_2_ (ref. 28), indicate that additional factors may cooperate to increase the sensitivity to AIs of TRPA1 expressing nociceptors. Enhancement by PAR2 activation of the proalgesic activity of exemestane and letrozole is fully consistent and closely mimic previous observations that PAR2 activation increases the pro-algesic response evoked by TRPA1 agonists^52^. Findings that a combination of AIs and H_2_O_2_ exaggerates TRPA1-mediated *in vitro* and *in vivo* responses suggest that increased levels of oxidative stress byproducts, known to be generated under inflammatory conditions^59^, may facilitate the development of AIMSS and related pain symptoms. Our present investigation on the cooperation between AIs and proinflammatory mediators has been limited to PAR2 and H_2_O_2_. However, it is possible that additional pro-inflammatory and pro-algesic mediators can activate similar cooperating pathways. AI concentrations required for TRPA1 activation are higher than those found in the plasma of treated subjects^60^,^61^,^62^. However, it should be noted that all three AIs have a large volume of distribution, indicating a high tissue distribution^49^,^51^. The present findings that in mice plasma levels of both AIs were comparable to those found in humans^49^,^51^ strengthen the hypothesis that compartmentalization of AIs in mice is similar to that reported in humans^49^,^51^. Thus, under standard drug regimens, concentrations sufficient to activate TRPA1 or to potentiate TRPA1-mediated responses evoked in cooperation with inflammatory mediators may be reached in tissues neighbouring sensory nerve terminals.

Altogether, the present results indicate that AIs *per se* or, most likely, in cooperation with other proinflammatory mediators promote TRPA1-dependent neurogenic inflammation, mechanical hypersensitivity and decreased forelimb grip force in rodents. This novel pathway may represent the main underlying mechanism responsible for pain and inflammatory symptoms associated with AI treatment. The other important proposal deriving from the present findings is that antagonists of the TRPA1 channel may be beneficial in the prevention and treatment of such painful conditions.

## Methods

### Animals

Animal experiments were carried out in conformity to the European Communities Council (ECC) guidelines for animal care procedures and the Italian legislation (DL 116/92) application of the ECC directive 86/609/EEC. Studies were conducted under the University of Florence research permit number 204/2012-B. Male C57BL/6 (25–30 g) (Harlan Laboratories, Milan, Italy), wild type, *Trpa1*^+/+^, or TRPA1-deficient, *Trpa1*^*−*/*−*^, (25–30 g) mice generated by heterozygous on a C57BL/6 background (B6;129P-Trpa1tm1Kykw/J; Jackson Laboratories, Italy)^63^, or Sprague–Dawley rats (75–100 g, male, Harlan Laboratories, Milan, Italy) were used. Animals were housed in a temperature- and humidity-controlled *vivarium* (12 h dark/light cycle, free access to food and water). Behavioural experiments were done in a quiet, temperature-controlled (20 to 22 °C) room between 0900 and 1700 hours and were performed by an operator blinded to the genotype and the drug treatment. Animals were killed with a high dose of sodium pentobarbital (200 mg kg^−1^, i.p.).

### Reagents

Exemestane, letrozole and anastrozole were purchased from Tocris Bioscience (Bristol, UK). The activating peptide (PAR2-AP, SLIGRL-NH_2_) and its reverse peptide (PAR2-RP, LRGILS-NH_2_) of the murine PAR2 receptor were synthesized from G. Cirino (University of Naples, Naples, Italy) and dissolved in distilled water. If not otherwise indicated, all other reagents were from Sigma-Aldrich (Milan, Italy). HC-030031 was synthesized as previously described^45^.

### Cell culture and isolation of primary sensory neurons

Human embryonic kidney (HEK293) cells stably transfected with the cDNA for human TRPA1 (hTRPA1-HEK293), kindly donated by A.H. Morice (University of Hull, Hull, UK) or with the cDNA for human TRPV1 (hTRPV1-HEK293), kindly donated by Martin J. Gunthorpe (GlaxoSmithKline, Harlow, UK) and naive untransfected HEK293 cells (American Type Culture Collection, Manassas, VA, USA) were cultured as previously described^64^. HEK293 cells were transiently transfected with the cDNAs (1 μg) codifying for wild-type or mutant 3C/K-Q (C619S, C639S, C663S, K708Q)^19^,^41^ human TRPA1 using the jetPRIME transfection reagent (Euroclone, Milan, Italy) according to the manufacturer’s protocol.

Primary DRG neurons were isolated from Sprague–Dawley rats and C57BL/6 or *Trpa1*^+/+^ and *Trpa1*^*−*/*−*^ adult mice, and cultured as previously described^38^. In brief, ganglia were bilaterally excised under a dissection microscope and enzymatically digested using 2 mg ml^−1^ of collagenase type 1A and 1 mg ml^−1^ of trypsin, for rat DRG neurons, or 1 mg ml^−1^ of papain, for mouse DRG neurons, in Hank’s Balanced Salt Solution (HBSS) for 25–35 min at 37 °C. Rat and mouse DRG neurons were pelleted and resuspended in Dulbecco’s Modified Eagle’s Medium (DMEM) supplemented with 10% heat inactivated horse serum or Ham’s-F12, respectively, containing 10% heat-inactivated fetal bovine serum (FBS), 100 U ml^−1^ of penicillin, 0.1 mg ml^−1^ of streptomycin and 2 mM L-glutamine for mechanical digestion. In this step, ganglia were disrupted by several passages through a series of syringe needles (23–25G). Neurons were then pelleted by centrifugation at 1,200 *g* for 5 min, suspended in *medium* enriched with 100 ng ml^−1^ mouse-NGF and 2.5 mM cytosine-b-D-arabino-furanoside free base, and then plated on 25 mm glass coverslips coated with poly-L-lysine (8.3 μM) and laminin (5 μM). DRG neurons were cultured for 3–4 days before being used for calcium imaging experiments.

### Calcium imaging assay

Intracellular calcium was measured in transfected and untransfected HEK293 cells or in DRG neurons, as previously reported^65^. Plated cells were loaded with 5 μM Fura-2AM-ester (Alexis Biochemicals, Lausen, Switzerland) added to the buffer solution (37 °C) containing the following (in mM): 2 CaCl_2_; 5.4 KCl; 0.4 MgSO_4_; 135 NaCl; 10 D-glucose; 10 HEPES and 0.1% bovine serum albumin at pH 7.4. After 40 min, cells were washed and transferred to a chamber on the stage of a Nikon Eclipse TE-2000U microscope for recording. Cells were excited alternatively at 340 and 380 nm to indicate relative intracellular calcium changes by the Ratio_340/380_ recorded with a dynamic image analysis system (Laboratory Automation 2.0, RCSoftware, Florence, Italy). Cells and neurons were exposed to exemestane, letrozole and anastrozole (1–300 μM), AITC (10–30 μM), menthol (100 μM), icilin (30 μM) or their vehicles (1.5–3% dimethyl sulfoxide, DMSO). The calcium response to capsaicin (0.1 μM) was used to identify nociceptive neurons. The selective TRPA1 antagonist, HC-030031 (30 μM), and TRPV1 antagonist, capsazepine (10 μM) or their vehicles (3% and 0.1% DMSO, respectively), were applied 10 min before the stimuli. Results are expressed as or the percentage of increase of Ratio_340/380_ over the baseline normalized to the maximum effect induced by ionomycin (5 μM) added at the end of each experiment (% change in R_340/380_) or Ratio_340/380_.

### Electrophysiology

Whole-cell patch-clamp recordings were performed on hTRPA1-HEK293, vector-HEK293 cells or rat DRG neurons grown on a poly-L-lysine-coated 13 mm-diameter glass coverslips. Each coverslip was transferred to a recording chamber (1 ml volume) mounted on the platform of an inverted microscope (Olympus CKX41, Milan, Italy) and superfused at a flow rate of 2 ml min^−1^ with a standard extracellular solution containing (in mM): 10 HEPES, 10 D-glucose, 147 NaCl, 4 KCl, 1 MgCl_2_ and 2 CaCl_2_ (pH adjusted to 7.4 with NaOH). Borosilicate glass electrodes (Harvard Apparatus, Holliston, MA, USA) were pulled with a Sutter Instruments puller (model P-87) to a final tip resistance of 4–7 MΩ. Pipette solution used for HEK293 cells contained (in mM): 134 K-gluconate, 10 KCl, 11 EGTA, 10 HEPES (pH adjusted to 7.4 with KOH). When recordings were performed on rat DRG neurons, 5 mM CaCl_2_ was present in the extracellular solution and pipette solution contained (in mM): CsCl 120, Mg_2_ATP 3, BAPTA 10, HEPES-Na 10 (pH adjusted to 7.4 with CsOH). Data were acquired with an Axopatch 200B amplifier (Axon Instruments, CA, USA), stored and analysed with a pClamp 9.2 software (Axon Instruments, CA, USA). All the experiments were carried out at 20–22 °C. Cells were voltage-clamped at –60 mV. Cell membrane capacitance was calculated in each cell throughout the experiment by integrating the capacitive currents elicited by a ±10 mV voltage pulse. In hTRPA1-HEK293 currents were detected as inward currents activated on cell superfusion with AITC (100 μM), exemestane (50–200 μM), letrozole (50–200 μM) or anastrozole (50–200 μM) in the presence of HC-030031 (50 μM) or its vehicle (0.5% DMSO). TRPV1 currents in rat DRG neurons were detected as inward currents activated by capsaicin (1 μM) in the presence of capsazepine (10 μM) or its vehicle (0.1% DMSO). To evaluate the potentiating effect of H_2_O_2_ or PAR2-AP on AI-activated currents, rat DRG neurons were superfused with H_2_O_2_ or PAR2-AP (both 100 μM) 1 min before and during the application of exemestane or letrozole (both, 20 μM). Some experiments were performed in the presence of HC-030031 (50 μM) or its vehicle (0.5% DMSO). Peak currents activated by each compound were normalized to cell membrane capacitance and expressed as mean of the current density (pA/pF) in averaged results. Currents were evoked in the voltage-clamp mode at a holding potential of −60 mV; signals were sampled at 1 kHz and low-pass filtered at 10 kHz.

### Behavioural experiments

For behavioural experiments, after habituation and baseline of pain sensitivity measurements, mice were randomized into treatment groups. In a first series of experiments, we explored whether the injection (20 μl per paw) of exemestane (1, 5, 10 nmol) or letrozole (10, 20 nmol), or their vehicle (5% DMSO) induced, in C57BL/6 or *Trpa1*^*+/+*^ and *Trpa1*^*−/−*^ mice, an acute nociceptive behaviour and a delayed mechanical allodynia. In this set of experiments mechanical allodynia was measured just before (30 min) and 0.25, 0.5, 1, 2, 4 and 6 h post injection. Some C57BL/6 mice were pretreated with HC-030031 (100 mg kg^−1^, i.p.) or capsazepine (10 mg kg^−1^, i.p.) or their respective vehicles (4% DMSO and 4% Tween20 in isotonic solution), 60 min and 30 min, respectively, before exemestane (10 nmol) or letrozole (20 nmol) i.pl. injection. Mechanical allodynia was measured 60 min after AIs i.pl. injection.

In a second set of experiments, nociceptive behaviour and mechanical allodynia were assayed before and after systemic administration of exemestane (5 mg kg^−1^, i.p. or 10 mg kg^−1^, i.g.) and letrozole (0.5 mg kg^−1^, i.p. or i.g.), or their vehicles (5% DMSO for i.p. or 0.5% carboxymethylcellulose, CMC, for i.g. administration), in C57BL/6 mice or *Trpa1*^*+/+*^ and *Trpa1*^*−/−*^ mice. Mechanical allodynia was measured just before (30 min) and 1, 3, 6, 24, 48 h after injection. Some animals 2 h after AI administration received HC-030031 (100 mg kg^−1^, i.p.) or its vehicle (4% DMSO and 4% Tween80 in isotonic solution), and mechanical allodynia and the forelimb grip strength were measured 1 and 3 h after vehicle or HC-030031. In a third series of experiments, *Trpa1*^*+/+*^ and *Trpa1*^*−/−*^ mice were treated i.p. once a day for 15 consecutive days with exemestane or letrozole at the dose of 5 or 0.5 mg kg^−1^, respectively, or with their vehicle (5% DMSO) and with i.g. exemestane or letrozole at the dose of 10 or 0.5 mg kg^−1^, respectively, or with their vehicle (0.5% CMC). Mechanical allodynia and the forelimb grip strength were measured 10 min before and 1, 3, 6 and 24 h post administration at day 1, 5, 10 and 15.

To test whether PAR2 activation enhances the nocifensor behaviour evoked by exemestane and letrozole, in another experimental setting, the PAR2 activating peptide (PAR2-AP), SLIGRL-NH_2_, (10 μg/10 μl i.pl.) or its reversed inactive form (PAR2-RP), LRGILS-NH_2_, (10 μg per 10 μl i.pl.), were injected in the right hind paw. Ten minutes after i.pl. PAR2-AP or PAR2-RP injection, mice received exemestane (10 nmol per 10 μl i.pl.) or letrozole (20 nmol per 10 μl, i.pl.), or their vehicle (5% DMSO), in the plantar surface in the same paw injected with PAR2-AP or PAR2-RP, and the acute nociceptive behaviour was recorded. In another series of experiments H_2_O_2_ (0.5 μmol per 10 μl, i.pl.) or its vehicle was injected and the acute nocifensor behaviour to H_2_O_2,_ which did not last longer than 5 min, was recorded for 10 min. Ten minutes after vehicle/H_2_O_2_, exemestane (10 nmol per 10 μl i.pl.) or letrozole (20 nmol per 10 μl, i.pl.) was injected in the same paw injected with H_2_O_2_ or vehicle and the acute nociceptive behaviour in response to AIs was recorded. Three hours after systemic administration of exemestane (5 mg kg^−1^, i.p.) or letrozole (0.5 mg kg^−1^, i.p.), mice were locally injected with H_2_O_2_ (0.5 μmol per 20 μl, i.pl.) or its vehicle and both acute nocifensor behaviour and mechanical allodynia were recorded.

*Acute nocifensive response*. AITC (10 nmol per paw), exemestane (10 nmol per paw), letrozole (20 nmol per paw) or their vehicles (5% DMSO), H_2_O_2_ (0.5 μmol per paw) or its vehicle (isotonic solution) and PAR2-AP or PAR2-RP (10 μg per paw) (10 or 20 μl) were injected into the paw of C57BL/6, *Trpa1*^*+/+*^ and *Trpa1*^*−/−*^ mice, and immediately after injection animals were placed in a plexiglas chamber. The total time spent licking and lifting the injected hind paw was recorded for 5 min as previously described^30^.

*Mechanical stimulation (von frey hair test)*. Mechanical threshold was measured in C57BL/6, *Trpa1*^*+/+*^ and *Trpa1*^*−/−*^ mice after both local (i.pl.) administration of AITC (10 nmol per paw), exemestane (10 nmol per paw), letrozole (20 nmol per paw) or their vehicles (5% DMSO), H_2_O_2_ (0.5 μmol per paw) or its vehicle (isotonic solution), and systemic (i.p.) administration of exemestane (5 mg kg^−1^, i.p.) or letrozole (0.5 mg kg^−1^, i.p.) at different time points by using the up-and-down paradigm^66^. Mechanical nociceptive threshold was determined before (basal level threshold) and after different treatments. The 50% mechanical paw withdrawal threshold response (in g) was then calculated from these scores, as previously described^66^,^67^.

*Forelimb grip strength test*. The grip strength test was performed with a grip strength meter (Ugo Basile, Varese, Italy), as previously reported^68^. Mice were allowed to grasp a triangular ring attached to a force transducer and gently pulled away by the base of the tail until the grip was broken. The test was repeated four times and the mean peak force values (g) were calculated for each animal. The grip strength was measured in C57BL/6, *Trpa1*^*+/+*^ and *Trpa1*^*−/−*^ mice 10 min before and 1, 3, 6 and 24 h post AI administration.

### Paw oedema

AITC (10 nmol per paw), exemestane (10 nmol per paw), letrozole (20 nmol per paw) or their vehicles (5% DMSO) (all 20 μl) were injected into the paw of C57BL/6, *Trpa1*^*+/+*^ and *Trpa1*^*−/−*^ mice, and paw thickness was measured to determine the development and severity of oedema in the hind paws. Some animals received HC-030031 (100 mg kg^−1^, i.p.), a combination of L-733,060 and CGRP8-37 (both, 2 μmol/kg, i.v.), or their vehicles (4% DMSO and 4% Tween20 in isotonic solution for HC-030031, and isotonic solution for L-733,060 and CGRP8-37) before stimuli. An engineer’s micrometer, with 0.01 mm accuracy (Harvard Apparatus, Kent, UK), was used to measure the paw thickness in millimeters (mm), before and after (60 and 120 min) the i.pl. injection with tested agents by an investigator blinded to treatments. Data were expressed as the increase in mm in paw thickness.

### CGRP-like immunoreactivity (LI) assay

For neuropeptide release experiments, 0.4 mm slices of rat and *Trpa1*^+/+^ or *Trpa1*^*−*/*−*^ mouse spinal cords were superfused with an aerated (95% O_2_ and 5% CO_2_) Krebs solution containing (in mM): 119 NaCl, 25 NaHCO_3_, 1.2 KH_2_PO_4_, 1.5 MgSO_4_, 2.5 CaCl_2_, 4.7 KCl, 11 D-glucose; the solution was maintained at 37 °C, and was added with 0.1% bovine serum albumin, and, to minimize peptide degradation, with the angiotensin converting enzyme inhibitor, captopril (1 μM), and the neutral endopeptidase inhibitor, phosphoramidon (1 μM). Tissues were stimulated with exemestane, letrozole or anastrozole (all 100 μM) or their vehicles (0.05% DMSO) dissolved in the Krebs solution. Some tissues were pre-exposed to capsaicin (10 μM, 20 min) or pretreated with HC-030031 (50 μM). Fractions (4 ml) of superfusate were collected at 10-min intervals before, during and after administration of the *stimulus* and then freeze-dried, reconstituted with assay buffer and analysed for CGRP-like immunoreactivity (LI) by an ELISA assay kit (Bertin Pharma, Montigny le Bretonneux, France). CGRP-LI was calculated by subtracting the mean *pre-stimulus* value from those obtained during or after stimulation. Detection limits of the assays were 5 pg ml^−1^. Results are expressed as femtomoles of peptide per g of tissue per 10 min.

In another set of experiments, exemestane (5 nmol per 50 μl) and letrozole (10 nmol per 50 μl) or their vehicle (1% DMSO) were i.a. injected in anaesthetized (sodium pentobarbital, 50 mg kg^−1^ i.p.) rats. Ten minutes after injection, rats were killed and the knee joint was dissected^69^. CGRP-LI was measured in the synovial fluid lavage added with captopril (1 μM) and phosphoramidon (1 μM) by using the ELISA assay kit as previously described^69^. Detection limits of the assays were 5 pg ml^−1^. Results are expressed as femtomoles of peptide per g of tissue per 20 min in the spinal cord experiments or pg ml^−1^ in the rat synovial fluid.

### Assay of exemestane and letrozole by liquid chromatography-mass spectrometry

Blood samples (100 μl) were obtained by venepuncture of the tail vein from each mouse at different time points (0.25, 0.5, 1, 3, 6 and 24 h) after i.g. administration of exemestane (10 mg kg^−1^) or letrozole (0.5 mg kg^−1^). Blood samples were dropped on a filter paper (903 Whatman GmbH, Dassel, Germany) to obtain dried blood spots (DBS)^70^, which were punched, obtaining a 6.0 mm diameter disk, containing ~6 μl of blood. DBS transferred into a 2-ml Eppendorf vial was extracted with 200 μl of methanol:water (95:5, v/v) containing 0.1% acetic acid and the appropriate internal standard (for letrozole and exemestane quantification, extracting solutions contained 5 μg l^−1^ of anastrozole or 2 μg l^−1^ of letrozole, respectively) and after shaking with an orbital shaker for 25 min at 37 °C, solutions were dried under a gentle nitrogen stream. Residues were reconstituted with 40 μl water containing 0.1% of acetic acid.

Samples were measured using a 1290 Infinity liquid chromatograph (LC, Agilent Technologies, Waldbronn, Germany) coupled to a QTRAP 5500 (AB SCIEX, Toronto, Canada) equipped with the Turbo Ion Spray source operating in positive ion mode. The capillary voltage was set to 5 kV. Heated turbo gas (400 °C, air) at a flow rate of 10.0 l min^−1^ was used. The transitions (quantifier and qualifier) recorded in Multiple Reaction Monitoring (MRM) mode were 286.1>217.1 and 286.1>190.1 for letrozole, 294.1>225.1 and 294.1>210.1 for anastrozole and 297.1>121.0 and 297.1>93.1 for exemestane. The LC column was a Gemini C6-Phenyl (100 × 2 mm^2^, 3 μm) with the corresponding 4 × 2 mm^2^ SecurityGuardTM cartridge (Phenomenex, Torrance, CA), operated at 0.3 ml min^−1^. Eluent A (water+0.1% acetic acid) and B (acetonitrile) were used. The gradient elution programme was as follows: 20% B maintained for 2 min, then to 90% B in 7 min, back to 20% B in 1 min and re-equilibrated for a 20 min total run time. Anastrozole, exemestane and letrozole retention times were 6.12, 6.31 and 7.45 min, respectively. Four microlitres of the extracted sample were injected for LC-MS/MS assays. System control and data acquisition were done by Analyst 1.5.1 software, and calibration curves were calculated using the non-weighted linear least-square regression of Analyst Quantitation programme (AB SCIEX, Toronto, Canada).

Calibration curves were constructed for both exemestane and letrozole, using the appropriate internal standard. Whole-blood from control mouse was spiked with different concentrations of exemestane (from 2 to 100 μg l^−1^) or letrozole (from 10 to 200 μg l^−1^). A 20 μl volume for each fortified blood sample was spotted on filter paper (DBS) and then treated as described in sample preparation. Each calibration curve was prepared in duplicate. Satisfying linearity was obtained for the two analytes (letrozole, *r*=0.996; exemestane, *r*=0.998). Each analytical batch included a double blank sample (without internal standard), a blank sample (with internal standard), five or six standard concentrations for calibration curve, and a set of treated mouse samples (each prepared in duplicate). LC-MS grade acetic acid, methanol, water and acetonitrile were supplied by Sigma Aldrich (Milan, Italy).

### Statistical analysis

Data represent mean±s.e.m. or confidence interval (CI). Statistical analysis was performed by the unpaired two-tailed Student’s *t*-test for comparisons between two groups, the ANOVA, followed by the Bonferroni *post-hoc* test for comparisons between multiple groups. Agonist potency was expressed as half maximal effective concentration (EC_50_), that is, the molar concentration of agonist producing 50% of the maximum measured effect and 95% confidence interval (CI). *P*<0.05 was considered statistically significant (GraphPadPrism version 5.00, San Diego, CA).

## Author contributions

C.F., R.N., S.M., P.G., S.B., M.R.D.T., T.S. and T.T. designed experiments, interpreted results and wrote the paper. C.F., S.M. and F.D.L. performed calcium experiments, E.C. performed electrophysiological experiments, R.N., I.M.M., G.T. and D.M. performed *in vivo* experiments, G.M. and G.P. performed mass spectrometry analyses.

## Additional information

**How to cite this article:** Fusi, C. *et al.* Steroidal and non-steroidal third-generation aromatase inhibitors induce pain-like symptoms via TRPA1. *Nat. Commun.* 5:5736 doi: 10.1038/ncomms6736 (2014).

## Supplementary Material

Supplementary Information Supplementary Figures 1-5
