# Supplementary material for: Steroidal and non-steroidal third-generation aromatase inhibitors induce pain-like symptoms via TRPA1
Source: Nat Commun. 2014 Dec 8;5:5736. doi: 10.1038/ncomms6736 (PMC4268712; doi:10.1038/ncomms6736)
Supplement: Supplementary Information — Supplementary Figures 1-5 [file ncomms6736-s1.pdf]

Supplementary information

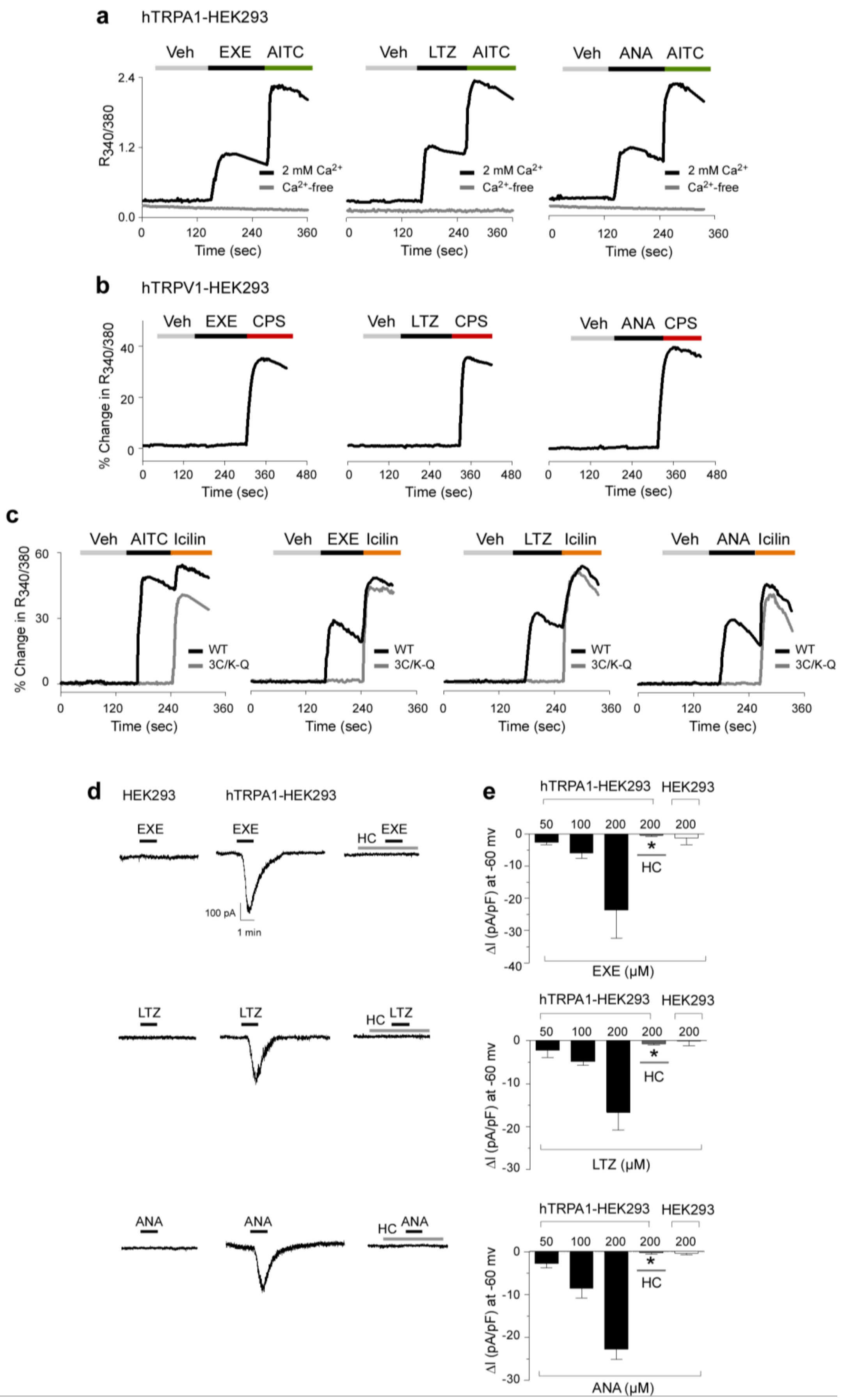

Supplementary Figure 1. Exemestane (EXE), letrozole (LTZ) and anastrozole (ANA) elicit a calcium influx and an inward current by selectively stimulating the human TRPA1 channel. (**a**) Representative traces of calcium mobilization evoked by EXE (100 µM), LTZ (100 µM), ANA (100 µM) in HEK293 cells stably transfected with the cDNA codifying for human TRPA1 (hTRPA1-HEK293) which also respond to allyl isothiocyanate (AITC; 30 µM). These effects are abated in a Ca^{2+}-free medium. Due to the absence of Ca^{2+} in the medium, results are expressed as $Ratio_{340/380}$ and not as % of increase of $Ratio_{340/380}$ normalized to ionomycin. (**b**) AIs (all 100 µM) fail to evoke any calcium response in HEK293 cells stably transfected with the cDNA codifying for human TRPV1 (hTRPV1-HEK293), which respond to the selective TRPV1 agonist, capsaicin (CPS; 0.1 µM). (**c**) Representative traces of cells transfected with the cDNA codifying for the mutant hTRPA1 channel (3C/K-Q), which are insensitive to AITC (30 µM) or AIs (100 µM), but respond to the non-electrophilic agonist, icilin (30 µM), whereas HEK293 cells transfected with the cDNA codifying for the wild type hTRPA1 (WT) respond to all the drugs. Veh is the vehicle of AIs. (**d**) Representative traces and (**e**) pooled data obtained by whole-cell patch-clamp recordings in hTRPA1-HEK293 cells. Exposure to EXE, LTZ or ANA elicits a concentration-dependent inward current at -60 mV in hTRPA1-HEK293, but not in untransfected (HEK293) cells. The selective TRPA1 antagonist, HC-030031 (HC; 50 µM), abolishes currents evoked by all the AIs. Results are mean ± s.e.m. of at least 5 cells tested for each experimental condition.*$P<0.05$ *vs*. EXE, LTZ or ANA; ANOVA and Bonferroni *post hoc* test.

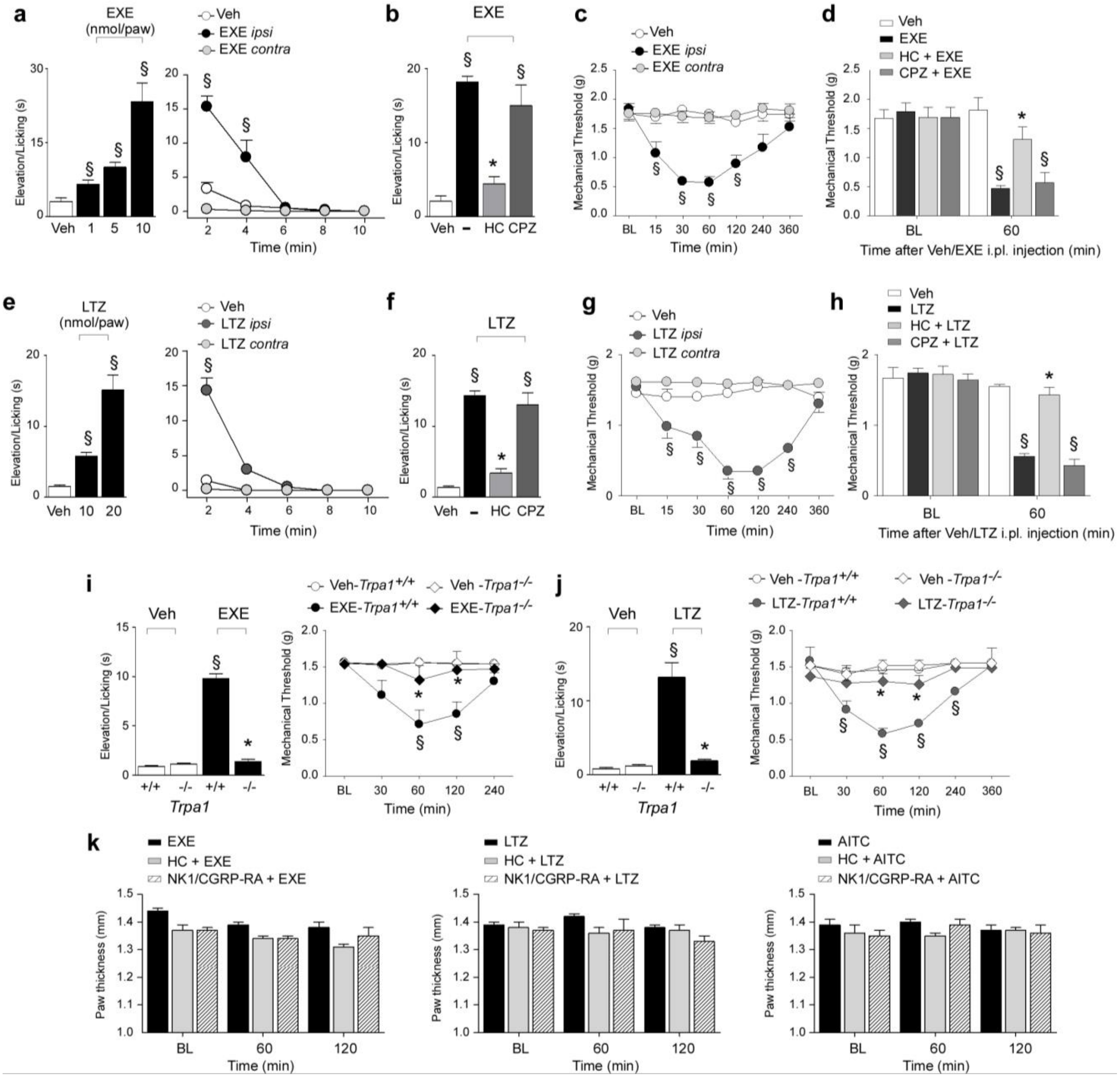

Supplementary Figure 2. **Local administration of exemestane (EXE) or letrozole (LTZ) induces acute nociception and mechanical allodynia *via* TRPA1 activation**. (**a**) Intraplantar (i.pl., 20 µl) injection of EXE (1-10 nmol) or (**e**) LTZ (10-20 nmol) in C57BL/6 mice induces pain-related behavior that lasts 5 minutes and is attenuated (**b**,**f**) by intraperitoneal (i.p.) HC-030031 (HC, 100 mg/kg), but not capsazepine (CPZ, 4 mg/kg, i.p.). (**c**) EXE (10 nmol) and (**g**) LTZ (20 nmol) produce mechanical allodynia that starts 15 minutes and lasts 120 or 240 minutes, respectively, after the i.pl. injection. (**d**,**h**) The mechanical allodynia induced by EXE- and LTZ (60 minutes after i.pl. injection) is prevented by systemic HC, but not by CPZ. Results represent mean ± s.e.m. of at least 5 mice for each group. Veh is the vehicle of EXE or LTZ, dash (-) indicates the combination of the

vehicles of HC and CPZ. §*P*<0.05 *vs.* Veh or BL values, **P*<0.05 *vs.* EXE, LTZ; ANOVA followed by Bonferroni *post hoc* test. (**i,j**) EXE (10 nmol) or LTZ (20 nmol) (both i.pl., 20 µl) produce acute nociception and mechanical allodynia in *Trpa1*$^{+/+}$ mice, which are markedly reduced in *Trpa1*$^{-/-}$ mice. Results are mean ± s.e.m. of at least 5 mice for each group. Veh is the vehicle of EXE or LTZ. §*P*<0.05 *vs.* Veh, **P*<0.05 *vs.* EXE- or LTZ- *Trpa1*$^{+/+}$; ANOVA and Bonferroni *post hoc* test. (**k**) Injection (20 µl, i.pl.) of EXE (10 nmol), LTZ (20 nmol) or allyl isothiocyanate (AITC, 10 nmol) in the ipsilateral side (right paw) does not induce paw edema in the contralateral side (left paw). In all conditions baseline levels (BL) were recorded 30 minutes before EXE, LTZ or AITC administration.

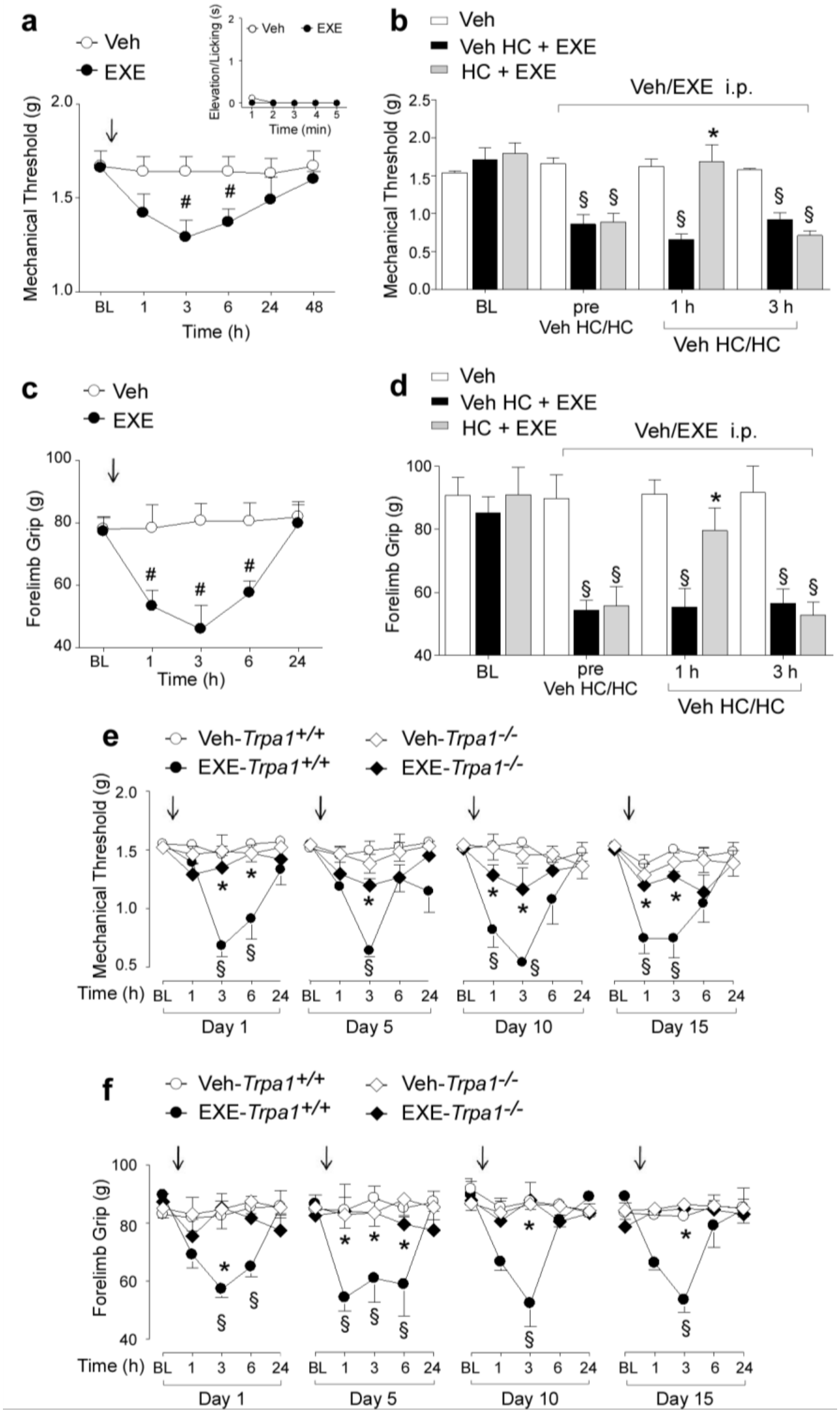

Supplementary Figure 3. Intraperitoneal exemestane (EXE) induces TRPA1-dependent prolonged mechanical allodynia and reduction in forelimb grip strength in mice. In C57BL/6 mice intraperitoneal (i.p.) administration of EXE (5 mg/kg) induces (**a**) mechanical allodynia and (**c**) a reduction in forelimb grip strength that last 3-6 hours after administration. EXE does not produce any acute nocifensor behavior as measured by the indicated test (**a,** inset). (**b,d**) Three hours after EXE administration, HC-030031 (HC; 100 mg/kg i.p.) reverts both mechanical

allodynia and reduction in forelimb grip strength. Inhibition is no longer visible 3 hours after its administration. Veh is the vehicle of EXE. $^{\#}P<0.05$ *vs.* Veh; Student's T test (**a,c)** and $^{\S}P<0.05$ *vs.* Veh and $^{*}P<0.05$ *vs.* Veh HC-EXE; ANOVA followed by Bonferroni *post hoc* test (**b,d**). (**e,f**) EXE (once a day for 15 consecutive days, 5 mg/kg i.p.) induces reproducible mechanical allodynia and decrease in grip strength at day 1, 5, 10 and 15 in *Trpa1*$^{+/+}$mice. Arrows indicate Veh or EXE administration. Both these effects are markedly reduced in *Trpa1*$^{-/-}$ mice. $^{\S}P<0.05$ *vs.* Veh-*Trpa1*$^{+/+}$, $^{*}P<0.05$ *vs* EXE-*Trpa1*$^{+/+}$; ANOVA followed by Bonferroni *post hoc* test. Results are mean ± s.e.m. of at least 5 mice for each group. In all conditions baseline levels (BL) were recorded 30 minutes before EXE administration.

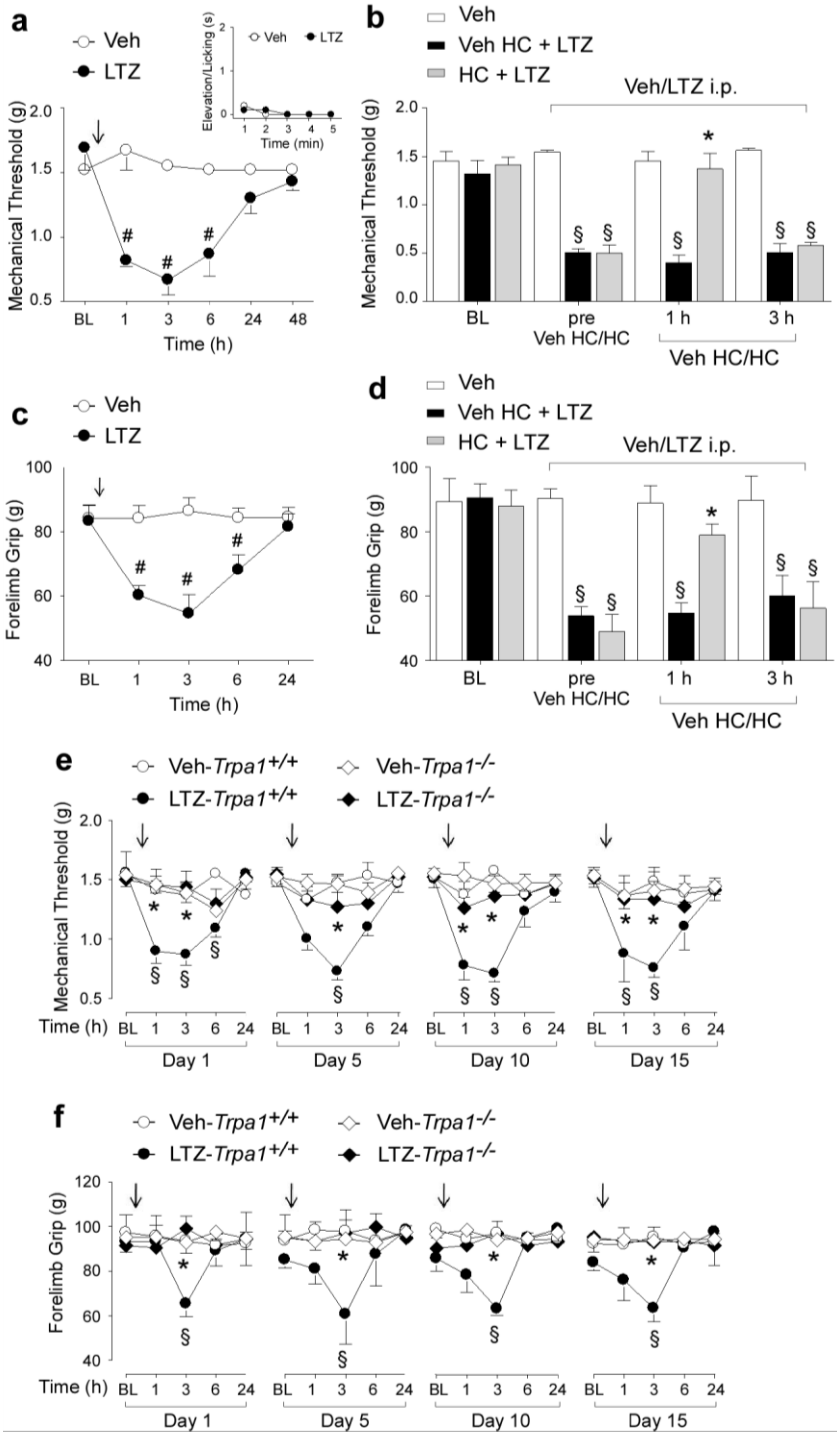

Supplementary Figure 4. Intraperitoneal letrozole (LTZ) induces TRPA1-dependent prolonged mechanical allodynia and reduction in forelimb grip strength in mice. Intraperitoneal (i.p.) administration of LTZ (0.5 mg/kg) induces (**a**) mechanical allodynia and (**c**) a reduction in forelimb grip strength that last 3-6 hours after administration in C57BL/6 mice. LTZ does not produce any acute nocifensor behavior as measured by the indicated test (**a,** inset). (**b,d**)

Three hours after LTZ administration HC-030031 (HC; 100 mg/kg i.p.) reverts both mechanical allodynia and reduction in forelimb grip strength. Inhibition by HC is no longer visible 3 hours after its administration. Veh is the vehicle of LTZ. $^{\#}P<0.05$ *vs.* Veh; Student's T test (**a,c)** and $^{§}P<0.05$ *vs.* Veh and $^{*}P<0.05$ *vs.* Veh HC-LTZ; ANOVA followed by Bonferroni *post hoc* test (**b,d**). (**e,f**) LTZ (once a day for 15 consecutive days, 0.5 mg/kg i.p.) induces reproducible mechanical allodynia and decrease in grip strength at day 1, 5, 10 and 15 in *Trpa1*$^{+/+}$mice. Arrows indicate Veh or LTZ administration. Both these effects are markedly reduced in *Trpa1*$^{-/-}$ mice. $^{§}P<0.05$ *vs.* Veh-*Trpa1*$^{+/+}$, $^{*}P<0.05$ *vs.* LTZ-*Trpa1*$^{+/+}$; ANOVA followed by Bonferroni *post hoc* test. Data are mean ± s.e.m. of at least 5 mice for each group. In all conditions baseline levels (BL) were recorded 30 minutes before LTZ administration.

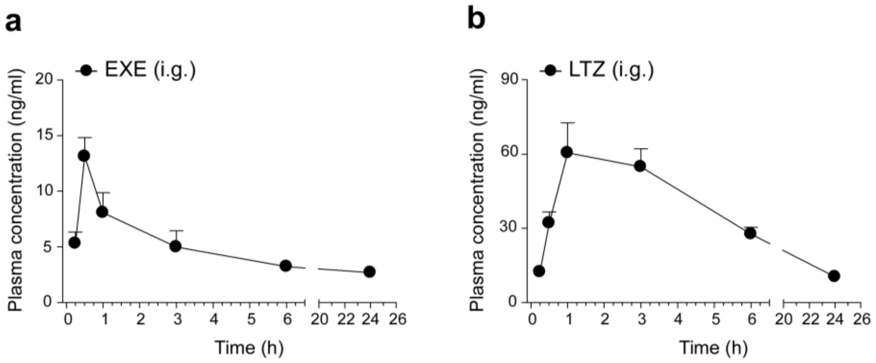

Supplementary Figure 5. Concentration-time profiles of exemestane (EXE) and letrozole (LTZ) in mouse plasma. Plasma concentrations measured at 0.25, 0.5, 1, 3, 6 and 24 hours after the intragastric (i.g.) administration of a single dose of EXE (10 mg/kg) or LTZ (0.5 mg/kg). Data are mean ± s.e.m. of at least 5 mice in each group.
